# Cross-Sectional Imaging of Pelvic Inflammatory Disease: Diagnostic Pearls and Pitfalls on CT and MR

**DOI:** 10.3390/diagnostics15162001

**Published:** 2025-08-10

**Authors:** Silvia Gigli, Marco Gennarini, Roberta Valerieva Ninkova, Valentina Miceli, Federica Curti, Sandrine Riccardi, Claudia Cutonilli, Flaminia Frezza, Chiara Amoroso, Carlo Catalano, Lucia Manganaro

**Affiliations:** 1 Department of Diagnostic Imaging, Sandro Pertini Hospital, Via dei Monti Tiburtini, 385, 00157 Rome, Italy; 2 Department of Radiological, Oncological and Pathological Sciences, Sapienza University of Rome, Viale Regina Elena 324, 00161 Rome, Italy; marco.gennarini@uniroma1.it (M.G.); robertavalerieva.ninkova@uniroma1.it (R.V.N.); valentina.miceli@uniroma1.it (V.M.); federica.curti@uniroma1.it (F.C.); sandrine.riccardi@uniroma1.it (S.R.); claudia.cutonilli@uniroma1.it (C.C.); flaminia.frezza@uniroma1.it (F.F.); chiara.amoroso@uniroma1.it (C.A.); carlo.catalano@uniroma1.it (C.C.); 3 Department of Experimental Medicine, Sapienza University of Rome, 00161 Rome, Italy

**Keywords:** pelvic inflammatory disease (PID), magnetic resonance imaging (MRI), computed tomography (CT), diffusion-weighted imaging (DWI), tubo-ovarian abscess (TOA)

## Abstract

Pelvic inflammatory disease (PID) encompasses a broad range of infection-induced inflammatory disorders of the female upper genital tract, commonly caused by ascending sexually transmitted infections. Diagnosis is often challenging because of nonspecific or absent symptoms and the overlap with other pelvic pathologies. While clinical and laboratory assessments are essential, cross-sectional imaging plays a pivotal role, especially in complicated, atypical, or equivocal cases. This review focuses on the typical and atypical imaging features of PID and highlights the crucial roles of computed tomography (CT) and magnetic resonance imaging (MRI) in its diagnostic evaluation. CT is frequently employed in emergency settings because of its widespread availability and ability to detect acute complications such as tubo-ovarian abscesses (TOA), peritonitis, or Fitz-Hugh–Curtis syndrome. However, it is limited by ionizing radiation and suboptimal soft-tissue contrast. MRI provides superior tissue characterization and multiplanar imaging without radiation exposure. When combined with diffusion-weighted imaging (DWI), MRI achieves high diagnostic accuracy, particularly in differentiating PID from other entities such as endometriosis, adnexal tumors, and gastrointestinal or urinary tract diseases. This review also addresses PID in specific clinical contexts, including post-partum infection, post-assisted reproductive technologies (ART), intrauterine device (IUD) use, and chronic or recurrent forms. A comprehensive, multimodal imaging approach integrated with clinical findings is essential for timely diagnosis, effective treatment, and prevention of severe reproductive sequelae.

## 1. Introduction

Pelvic inflammatory disease (PID) is an infection-induced inflammation affecting the upper female genital tract. The infection typically ascends from the lower genital tract and can lead to conditions such as endometritis, salpingitis, parametritis, tubo-ovarian abscess (TOA), and pelvic peritonitis. Young women under 25 years of age face a higher likelihood of developing PID. Most cases are caused by sexually transmitted infections (STIs), notably Chlamydia trachomatis, Neisseria gonorrhoeae, and Mycoplasma genitalium [1]. Approximately 15% of untreated chlamydial infections progress to PID, and this percentage may be higher with gonococcal infections. Other organisms involved can include Mycobacterium tuberculosis and a wide variety of facultative and anaerobic bacteria [2]. PID continues to be a significant public health burden. Based on the National Health and Nutrition Examination Survey (2013–2014), an estimated 4.4% of sexually experienced women aged 18–44 in the United States (approximately 2.5 million women) reported a lifetime history of PID [3]. Diagnosing PID is primarily based on clinical evaluation and can be challenging, since symptoms are often nonspecific and can range from being entirely absent (asymptomatic) to causing serious illness. Early diagnosis and prompt treatment are vital to potentially prevent short-term complications, such as tubo-ovarian or pelvic abscesses.

Delayed diagnosis of pelvic inflammatory disease can lead to serious long-term consequences, including infertility, chronic pelvic pain, and an increased risk of ectopic pregnancy [4] because of potential damage to the reproductive organs. 

Cross-sectional imaging, such as CT or MRI, is used in the diagnostic pathway for pelvic inflammatory disease (PID), providing more detailed anatomical information, particularly when the diagnosis is uncertain, when complications are suspected, or when the disease is chronic or complex. In addition, in emergency settings, cross-sectional imaging can play a crucial role in differentiating PID from other conditions. 

This review provides an up-to-date, evidence-based synthesis of current imaging strategies in PID, particularly focusing on cross-sectional imaging—specifically CT and MRI—which remains underrepresented in the current literature on PID. By integrating imaging findings with clinical scenarios, we provide practical guidance for radiologists and clinicians in evaluating both typical and complex diagnoses. Particular attention is given to differential diagnosis, providing key imaging features that distinguish PID from other pelvic pathologies.

## 2. Imaging

Transvaginal ultrasound (TVUS) is widely recognized as the first-line imaging modality for evaluating suspected PID, particularly in outpatient and emergency settings. It is a rapid, non-invasive, and cost-effective technique that offers broad accessibility and generally good patient tolerability. High-frequency transvaginal probes (5–9 MHz) provide excellent spatial resolution, allowing systematic assessment of the uterus, adnexa, posterior cul-de-sac, and parametrial tissues. The integration of power or color Doppler further facilitates the identification of inflammatory hyperemia [5,6]. In cases of suspected TOA, three-dimensional ultrasound may aid in characterizing complex adnexal masses and distinguishing between inflammatory and neoplastic processes.

However, the sensitivity of TVUS may be limited in the early stages of PID or patients with altered pelvic anatomy because of adhesions, obesity, or prior surgery, emphasizing the role of a multimodal imaging approach in selected cases. Despite its advantages, TVUS has intrinsic limitations. Patient intolerance, particularly in acute inflammatory settings, can result in significant discomfort, potentially compromising the feasibility and completeness of the examination. Additional limitations include a restricted field of view, limited tissue penetration, and high dependency on operator skill. Image quality may also be degraded by suboptimal bladder filling, bowel gas, or limited patient cooperation. Although studies comparing ultrasound to laparoscopy—the current gold standard—generally report good concordance, they often involve patients with clinically advanced disease, possibly leading to overestimation of diagnostic accuracy.

In more complex or ambiguous cases, second-line imaging techniques such as computed tomography (CT) or magnetic resonance imaging (MRI) are often required. CT plays an important role in the emergency setting, particularly when patients present with nonspecific lower abdominal pain or when ultrasound findings are inconclusive. CT offers excellent anatomic resolution and can reveal key features of acute PID, including enhancement in hepatic capsular thickening, enhancing fallopian tubes, pelvic fat stranding, uterine enlargement, endometrial enhancement, and complex adnexal masses suggestive of TOA [7,8]. The advent of multidetector computed tomography (MDCT), with thinner collimation, faster acquisition times, and improved spatial resolution, has further increased the diagnostic yield of CT for pelvic pathologies [9]. However, despite these technological advancements, CT is not recommended as a first-line modality because of radiation exposure and limited soft-tissue contrast. CT shows moderate-to-high sensitivity and specificity for PID, although most studies are retrospective and involve small sample sizes. The available literature on CT in PID consists primarily of descriptive or multimodality studies, with few large-scale prospective investigations [10,11].

MRI, by contrast, provides superior soft-tissue contrast, multiplanar capability, and avoids ionizing radiation. In cases where ionizing radiation is a concern—such as in adolescents or women of reproductive age—MRI serves as a safer alternative to CT, without compromising diagnostic accuracy. MRI should also be considered when ultrasound findings are inconclusive or when precise mapping of pelvic anatomy is essential for surgical planning or conservative management.

MRI demonstrated a sensitivity of 95% and specificity of 89% compared with laparoscopy, confirming its high diagnostic accuracy. Another study reported similar results with an overall diagnostic accuracy of 93% in complex cases when MRI was compared with TVUS [12,13]. The MRI protocol typically includes T1- and T2-weighted sequences in multiple planes, fat-suppressed images, and post-contrast imaging with gadolinium-based agents. The addition of diffusion-weighted imaging (DWI) has further enhanced the diagnostic performance of MRI. The inclusion of DWI significantly improves the detection of abscesses and areas of infection, increasing sensitivity and overall accuracy. In their study, MRI without DWI yielded a sensitivity of 90.7% and overall accuracy of 91.2%, whereas MRI with DWI achieved values of 98.4% and 97.5%, respectively [14].

Although MRI is not routinely used as a first-line tool because of its higher costs and lower availability, it plays a pivotal role in clinical decision-making when ultrasound and CT findings are inconclusive, often reducing the need for diagnostic laparoscopy and improving patient management [Table 1].

The critical roles of CT and MRI in the diagnostic evaluation of both typical and atypical imaging features of PID are summarized in Table 2.

## 3. Acute PID

### 3.1. Cervicitis and Endometritis

Cervicitis and endometritis are the earliest manifestations of PID. Cervicitis is inflammation of the uterine cervix. Clinically, it may be asymptomatic or associated with nonspecific symptoms such as pelvic discomfort, dyspareunia, spotting after intercourse, or a yellowish mucopurulent vaginal discharge. These symptoms result from mucosal irritation and vascular fragility within the infected cervical tissue. Cervicitis is broadly categorized into infectious and non-infectious forms. The ectocervix, with epithelium closely resembling the vagina, is most susceptible to the microorganisms *Trichomonas vaginalis*, *Candida albicans*, and herpes simplex virus (especially type 2), as well as those more generically responsible for bacterial vaginosis. *Neisseria gonorrhoeae* and *Chlamydia trachomatis* have a predilection for infecting the glandular epithelium of the endocervix [11,15].

Endometritis refers to the inflammation of the endometrial lining. In obstetric settings, it frequently arises in the post-partum period or following miscarriage (e.g., curettage). In non-obstetric populations, acute endometritis is typically associated with PID or invasive gynecologic procedures. Endometritis often coexists with cervicitis and, similar to it, may be subclinical. When symptomatic, it may be present with fever, lower abdominal pain, abnormal uterine bleeding, and leukocytosis [11,16].

There is limited coverage of these relatively common conditions in the imaging literature, likely because patients with cervicitis and endometritis are not routinely referred to imaging.

Ultrasound is the first-line imaging for suspected PID. Cervicitis may show cervical enlargement, stromal edema, cystic changes, and increased vascularity on Doppler [17]. Endometritis appears as a thickened, heterogeneous endometrium, intracavitary fluid or debris, loss of the normal endometrial–myometrial junction, and increased blood flow. Intrauterine gas (echogenic foci with shadowing) suggests anaerobic infection [18]. Free fluid in the pouch of Douglas may also be present.

**Figure 1:**
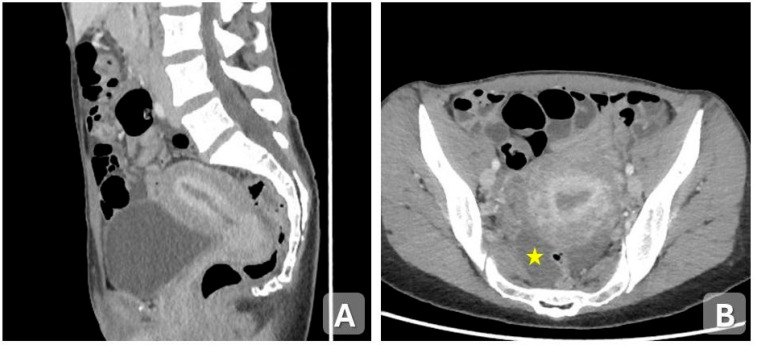
Acute endometritis in a 60-year-old woman with pelvic pain and a low-grade fever for 5 days. (**A**) Axial and (**B**) sagittal contrast-enhanced CT images show an enlarged, globular uterus with distension of the endometrial cavity containing hypodense fluid. There is enhancement of the endometrial lining after contrast administration. A large amount of free pelvic fluid is present (star).

CT is mainly used in emergencies. Cervicitis shows cervical wall thickening and fat stranding; healing may cause fibrosis and stenosis [7]. Endometritis may show an enlarged uterus with thickened endometrium, low-density fluid, gas, free pelvic fluid, and lymphadenopathy [7] [Figure 1].

**Figure 2:**
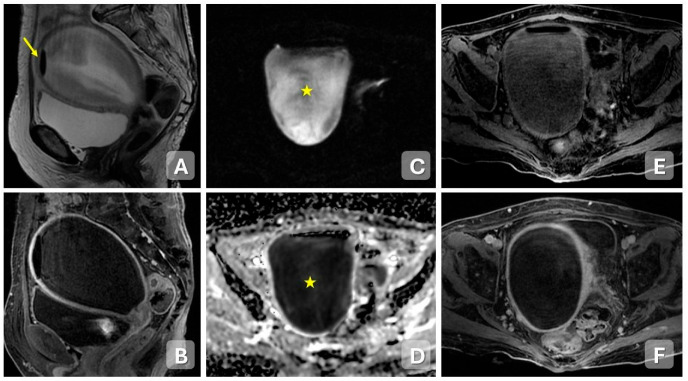
Acute pelvic inflammatory disease—endometritis in a 60-year-old woman with pelvic pain. (**A**,**B**) Sagittal T2-weighted and contrast-enhanced T1-weighted MRI images show a distended endometrial cavity filled with fluid containing corpuscular material, and a gas bubble (arrow) at the uterine fundus. Thinning of the myometrium and loss of the junctional zone are also observed. (**C**,**D**) DWI and ADC maps demonstrate restricted diffusion (star) within the endometrial cavity, consistent with purulent content. (**E**,**F**) Axial fat-saturated T1-weighted images before and after contrast administration show marked post-contrast enhancement of the endometrial lining.

MRI offers superior soft-tissue detail and is particularly useful when the diagnosis is equivocal. The normal endocervix and endometrium are T2 hyperintense relative to the underlying junctional zone and myometrium, which are markedly T2 hypointense, similar to normal smooth muscle at other anatomic sites. MRI has excellent sensitivity in demonstrating inflammation and edema on fat-suppressed T2-weighted sequences, which manifests as increased T2 signal in the cervical and uterine myometrium [13]. Inflamed cervix and endometrium show T2 hyperintensity and thickening, with loss of the normal junctional zone in endometritis. Gas appears as signal voids with susceptibility artifact (“blooming”) on T1 and gradient echo images. Contrast enhancement highlights inflamed areas. Importantly, MRI can help differentiate infectious cervicitis from malignancy. Unlike neoplastic lesions, infectious cervicitis generally lacks a solid mass or nodular enhancing components, and the cervical contour remains preserved [19] [Figure 2].

### 3.2. Salpingitis

Salpingitis—the inflammation of the fallopian tubes—is typically the earliest and most common clinical manifestation of PID, mainly affecting young, sexually active women. It carries a high risk of infertility and ectopic pregnancy. Often, endometritis coexists with salpingitis in 70–90% of cases, highlighting the complex and often overlapping nature of pelvic infections [2].

In acute salpingitis, the fallopian tubes become swollen, congested, and inflamed. As the condition worsens, pus (pyosalpinx) fills the tubal lumen and may leak into the peritoneal cavity, causing widespread inflammation. This can involve the peritoneum, coat nearby pelvic organs, and form adhesions. The fimbrial ends may stick to the ovary, causing salpingo-oophoritis or a tubo-ovarian complex, which can progress to a TOA if untreated [20]. Clinical symptoms vary, ranging from asymptomatic to severe pelvic pain, and do not always reflect severity.

In cases of salpingitis, the tubal walls become thickened, often exceeding 5 mm in diameter, and may appear tortuous, showing a coiled appearance, especially in cases of acute infection where the tubes are distended because of fluid accumulation. This tortuous pattern can be an early indicator of salpingitis. A hydrosalpinx, the most common form of fallopian tube distention, appears as a fluid-filled, non-purulent tube. The ultrasound typically reveals a hypo-anechoic (dark) tube without thickened walls. It is important to note that hydrosalpinx is typically a result of chronic PID and is less commonly seen in acute salpingitis. On MRI, the content of the tube appears hypointense on T1-weighted and hyperintense on T2-weighted sequences, without surrounding edema or wall enhancement [21,22].

In cases of pyosalpinx, the ultrasound image will show a distended fallopian tube filled with echogenic fluid. The presence of echogenic debris (pus) in the tube results in a heterogeneous echotexture inside the tube, which is characteristic of pus-filled tubes. One of the advantages of ultrasound is the ability to use color Doppler imaging, which can help evaluate vascular changes associated with inflammation in salpingitis. Inflammatory changes in the fallopian tubes and surrounding structures often result in increased blood flow. Color Doppler imaging can reveal increased vascularity within the tube wall, which suggests active inflammation [22,23].

In more severe cases of salpingitis, the fluid collection within the pelvis may become more complex, with debris or clot formation. Specific key signs are used in ultrasound to help identify salpingitis and distinguish it from other conditions. The cogwheel sign refers to the appearance of the fallopian tube when it is distended and shows internal mucosal folds that resemble a cogwheel or gear-like pattern. This is seen when the tube is filled with pus (pyosalpinx), and the folds of the tube’s internal mucosa become more discernible. Another characteristic sign of pyosalpinx is the beads on a string sign, where the distended tube shows a series of echoic areas within it, resembling beads on a string. The beads correspond to areas of mucosal folds or debris within the pus-filled tube, and they are a hallmark of infected tubes [23].

On MRI, pyosalpinx has mixed T1 and T2 signal intensities, depending on protein and cellular content. Wall enhancement and surrounding fat stranding are typically present. However, hemosalpinx appears as a T1 hyperintense, T2 hypointense fluid-filled tube, consistent with blood products. Hematosalpinx can be seen in cases where there is bleeding from the inflamed tube, but this condition is less commonly associated with PID and more often suggests endometriosis [24,25].

DWI is particularly useful in cases of pyosalpinx, as the purulent material typically exhibits restricted diffusion because of its high cellularity and viscosity [26]. Li et al. evaluated the diagnostic value of DWI when added to conventional MRI for detecting PID. The authors found that combining DWI with standard MRI significantly improved the accuracy of PID diagnosis [14].

The use of gadolinium-based contrast agents on MRI can provide further information about the vascularity and extent of inflammation. In inflamed fallopian tubes, the wall may demonstrate contrast enhancement, reflecting hyperemia and increased vascularity. This is particularly true in acute phases of salpingitis or in cases where there is active infection. In advanced salpingitis, contrast enhancement may be seen in the surrounding structures, such as the uterosacral ligaments, parametrial tissues, and pelvic fascia. This can suggest the spread of infection or the involvement of the broader pelvic structures [13].

**Figure 3:**
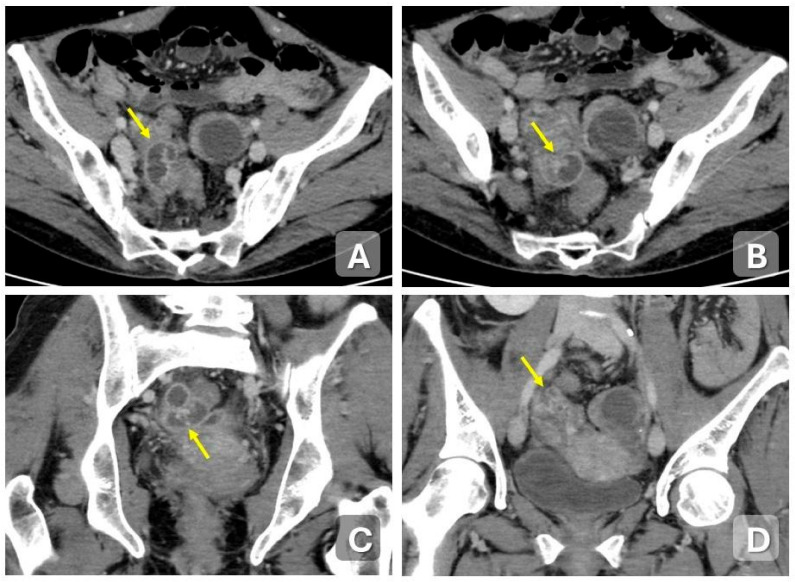
Acute pelvic inflammatory disease: salpingitis in a 27-year-old woman presenting with pelvic pain. Axial (**A**,**B**) and coronal (**C**,**D**) contrast-enhanced CT images demonstrate a distended fallopian tube with thickened, tortuous walls, exhibiting a characteristic coiled appearance (arrows).

CT is not the primary modality for diagnosing salpingitis, but it is often performed in the emergency setting when evaluating complications such as abscess formation, ileus, or bowel involvement. Importantly, CT may also help differentiate salpingitis from mimics such as appendicitis or diverticulitis, which can cause secondary tubal inflammation [27] [Figure 3].

### 3.3. Oophoritis, Tubo-Ovarian Complex (TOC), and Tubo-Ovarian Abscess (TOA)

Oophoritis may result from a direct infection of the ovary or as part of a broader inflammatory process involving the fallopian tubes, leading to tubo-ovarian complex (TOC) or tubo-ovarian abscess (TOA). It is important to differentiate oophoritis from other ovarian pathologies, such as ovarian cysts, neoplasms, or endometriomas, as the management and treatment approaches for these conditions differ significantly [28]. Clinically, oophoritis can present symptoms ranging from pelvic pain, fever, and abnormal vaginal discharge to more severe signs such as nausea, vomiting, and sepsis in the case of TOA. The clinical manifestations, however, can be nonspecific, and imaging is often required to confirm the diagnosis [29].

Inflammation leads to ovarian swelling or enlargement. The ovaries may appear larger than normal because of edema or congestion. On TVUS, ovarian parenchyma may become hypoechoic or heterogeneous because of the presence of edema, inflammatory cells, and sometimes pus. This change in echotexture is indicative of an active inflammatory process [30]. On MRI, the inflamed ovary appears enlarged, with a heterogeneous signal on T1 and T2-weighted images. The inflammatory changes may be more pronounced on fat-suppressed sequences, which show areas of edema or fluid collection as regions of increased signal intensity [25].

The ovary may be involved in a tubo-ovarian complex, where the ovary and fallopian tube are adherent but remain largely separate. A tubo-ovarian abscess is a severe and late complication of acute salpingitis, developing in up to 15% of patients with PID. It represents an advanced stage of infection, where the ovary and fallopian tube become indistinguishable because of widespread inflammation and tissue destruction. Clinically, TOAs may present similarly to uncomplicated PID—with pelvic pain, fever, vaginal discharge, and leukocytosis—though systemic signs may sometimes be absent. Hospitalization is frequently required, with TOAs being unilateral in 25–50% of cases [25,30].

TOAs are often complex masses with both cystic and solid components, reflecting necrosis and purulent material. Ultrasound will reveal a complex mass with heterogeneous echotexture, and sometimes hyperechoic foci representing gas or debris. Septations may be seen within the abscess cavity. On color Doppler imaging, increased vascularity may be noted in the ovary, indicating hyperemia associated with inflammation. In cases of TOA, MRI is particularly useful in delineating the extent and nature of the abscess. A well-defined fluid collection with enhanced walls can be seen, and the abscess may show hyperintensity on T1-weighted imaging if it contains hemorrhagic or proteinaceous material [13]. On T2-weighted imaging, the abscess typically appears as a hypointense structure. The ovaries may show hyperenhancement, which is a direct sign of active inflammation and hyperemia. Anterior displacement of the broad ligament may help identify TOA, as the mesovarium is posterior in origin. This displacement favors a tubo-ovarian origin. Thickened uterosacral ligaments reflect deep pelvic inflammation. Other findings include fat stranding, enhancement of pelvic fascia, and free fluid in the pouch of Douglas [31,32] [Table 3].

**Figure 4:**
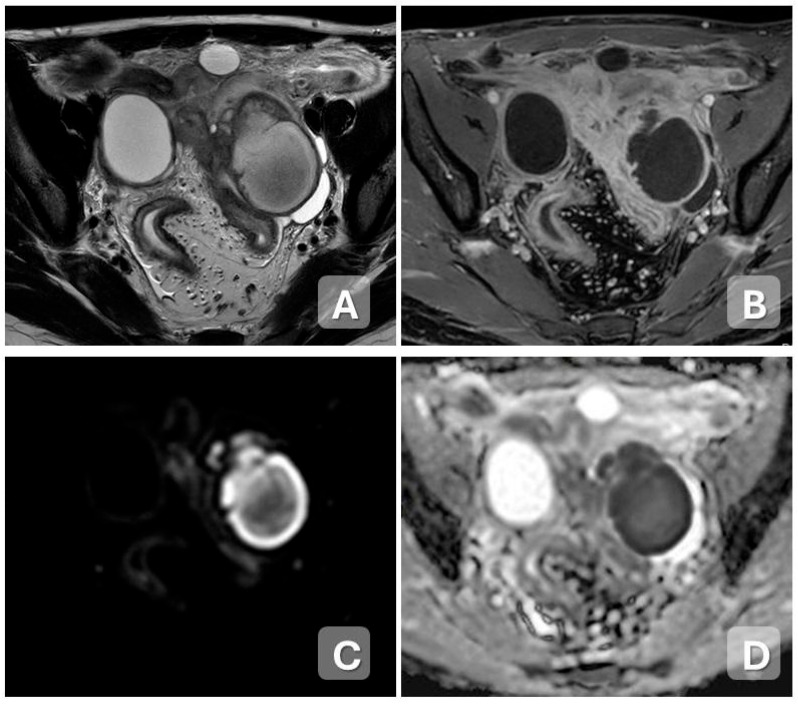
Tubo-ovarian abscess in a 30-year-old woman with worsening pelvic pain. An increase in volume of the left adnexa and left Fallopian tube, due to the presence of a formation with fluid-corpuscular content (**A**), showing restriction in DWI (**C**) and ADC (**D**), and homogeneous post-contrast graphic enhancement of the walls of the formation (**B**).

CT is generally not the first-line modality for diagnosing oophoritis but is often utilized in the emergency setting or when complications, such as abscess formation or peritonitis, or in suspected TOA rupture or extra-pelvic spread. CT provides detailed information about the extent of pelvic involvement and can help detect TOAs or peri-ovarian fluid collections. The abscesses typically demonstrate a complex appearance, with both hypodense (fluid-filled) and hyperdense (debris or hemorrhage) components. Gas bubbles within the mass are often seen in anaerobic infections or if the abscess is communicating with the bowel or ruptured [33,34] [Figure 4].

### 3.4. Peritonitis

PID can lead to peritonitis when infection spreads to the peritoneal cavity. Diagnosis relies mainly on CT, ultrasound, and sometimes MRI [11,35].

**Figure 5:**
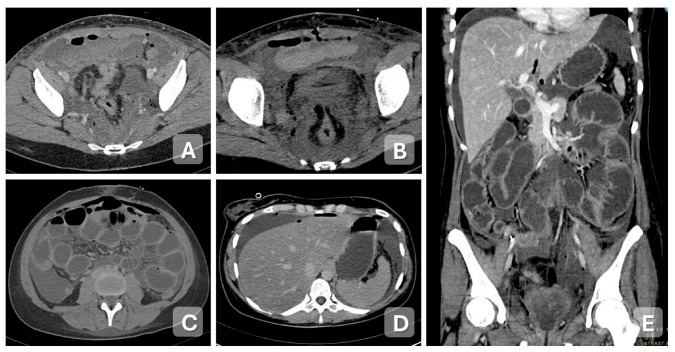
Pelvic inflammatory disease complicated with peritonitis: axial CT with contrast enhancement (**A**,**C**,**D**) and without (**B**) and coronal (**D**,**E**) CT with contrast enhancement showing fluid collections, enhanced peritoneal lining, fat stranding, inflamed bowel loops, pneumoperitoneum, and fatty infiltration around the bowel.

**Figure 6:**
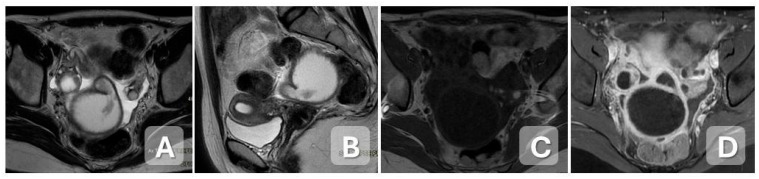
Pelvic inflammatory disease with peritonitis in a 31-year-old woman with a history of endometriosis and recent oocyte retrieval, presenting with pelvic pain and fever 5 days post-procedure. (**A**,**B**) Axial and sagittal T2-weighted MR images show a serpiginous left adnexal lesion with corpuscular fluid content, consistent with a dilated and inflamed fallopian tube. (**C**,**D**) Axial T1-weighted images before contrast administration and T1-weighted with fat saturation after contrast administration demonstrate marked enhancement of the thickened tubal walls. Associated free pelvic fluid and inflammatory stranding are consistent with secondary peritonitis.

Imaging findings include complex or loculated fluid collections, enhanced peritoneal lining, especially in pelvic and abdominal areas, and fat stranding around pelvic organs such as the uterus, ovaries, and fallopian tubes [36,37]. Inflamed bowel loops may be displaced, with possible bowel wall thickening, pneumoperitoneum, or fatty infiltration around the bowel, indicating severe or complicated peritonitis [35] [Figure 5 and Figure 6].

### 3.5. Fitz-Hugh–Curtis Syndrome

Fitz-Hugh–Curtis syndrome (FHCS) is a rare but important complication of PID, involving inflammation of the liver capsule (perihepatitis) and adhesions [38]. It results from ascending infection, mainly *Chlamydia trachomatis* or *Neisseria gonorrhoeae*, spreading hematogenously or via the peritoneum. FHCS occurs in 4–14% of PID cases, rising to 27% in adolescents [38]. Patients typically present with acute right upper quadrant pain, sometimes radiating to the shoulder, often without pelvic symptoms, complicating diagnosis without imaging [39].

Radiologic signs include hepatic capsule thickening and enhancement, loculated perihepatic fluid, gallbladder wall thickening, fat stranding, and fluid tracking from the pelvis to the right upper quadrant along the paracolic gutter [40,41]. These findings should raise suspicion for FHCS in young women with current or past PID, even if pelvic signs are minimal.

### 3.6. PID After ART

The use of assisted reproductive technologies (ART) has increased steadily with a corresponding increase in the number of ART-related procedures, such as hysterosalpingography, saline infusion sonography, hysteroscopy, laparoscopy, oocyte retrieval, and embryo transfer [42]. While performing these procedures, the abdomen, upper vagina, and endocervix are breached, leading to the possibility of seeding pelvic structures with microorganisms. The overall risk of such a procedural infection is rare, with an estimated incidence of 0.03% to 0.58% after transvaginal oocyte retrieval and lower after embryo transfer [43]. Risk factors for developing PID post-TVOR include: multiple IVF cycles, presence of endometriosis, and elevated pre-treatment C-reactive protein levels [44]. PID following ART can adversely affect fertility outcomes [45].

Imaging is crucial in this setting for assessing the infection’s extent, guiding timely intervention, and reducing risks to health and fertility. In TOA post-ART, ultrasound- or CT-guided drainage helps prevent sepsis [44]. It also allows for the assessment of treatment response, with serial imaging often needed to confirm resolution after antibiotics or surgery.

### 3.7. PID After Pregnancy and Delivery

PID after pregnancy, though less commonly discussed in the literature, is a potential post-partum complication. It typically results from ascending infections following vaginal delivery, cesarean section, or miscarriage. Causative organisms often include endogenous flora such as *Escherichia coli*, Group B Streptococcus, and *Staphylococcus aureus*, though STIs such as *Chlamydia trachomatis* and *Neisseria gonorrhoeae* may also contribute [46].

Incidence is estimated at 1–3% within six weeks post-partum, especially with risk factors such as prolonged labor, instrumentation, cesarean delivery, or incomplete miscarriage [47].

Imaging is essential in diagnosing PID, and complications such as TOAs can occur. Early diagnosis and timely treatment can prevent long-term reproductive harm. Notably, post-partum PID may increase the risk of uterine rupture after cesarean section—a rare but life-threatening event [48].

### 3.8. PID and Retained Products of Conception (RPOC)

PID and retained products of conception (RPOC) are important complications following miscarriage or induced abortion, and they may be interrelated. RPOC refers to persistent fetal or placental tissue in the uterus due to incomplete abortion or spontaneous miscarriage. This retained tissue provides a nutrient-rich environment for bacterial growth, potentially leading to endometritis and progressing to PID if untreated [49].

Endometritis is the most common post-abortion infection, and if the infection ascends, it may involve the fallopian tubes and ovaries, resulting in more severe PID [50]. RPOC is often detected on ultrasound as echogenic intrauterine material, endometrial thickening, and uterine enlargement. In complicated cases, infected RPOC may form abscesses or cavitary masses [51].

### 3.9. PID and IUD

Intrauterine devices (IUDs) are widely used long-acting reversible contraceptives. Although generally safe, IUD use carries a slightly increased risk of PID, especially within the first few weeks post-insertion [52]. While the overall incidence is low, PID in IUD users can lead to serious reproductive complications if left untreated [53]. The pathogenesis involves bacterial introduction during insertion or an ascending infection, especially in women with existing STIs or high-risk sexual behavior.

Ultrasound is essential for assessing IUD position and identifying PID-related complications. A correctly placed IUD appears as a highly echogenic linear structure in the endometrial cavity. Imaging can reveal malposition, embedment, or perforation [54]. Early diagnosis and management are vital to prevent chronic pelvic pain or infertility [55].

## 4. Chronic PID

Chronic PID is a consequence of unresolved or recurrent infection in the female upper genital tract and is a major contributor to long-term gynecologic morbidity. It commonly results from subclinical or inadequately treated episodes of acute PID, particularly infections due to *Chlamydia trachomatis*, *Neisseria gonorrhoeae*, *Mycoplasma genitalium*, and anaerobic bacteria associated with bacterial vaginosis [56,57]. Over time, sustained inflammation leads to irreversible structural damage, including fallopian tube scarring, tubo-ovarian distortion, and pelvic adhesions [58,59]. Pelvic adhesions are a hallmark of chronic PID and result from repeated cycles of inflammation, tissue necrosis, and fibroblast-mediated repair [19]. These adhesions can affect the uterus, ovaries, fallopian tubes, bowel, and peritoneum, leading to chronic pelvic pain, dyspareunia, and infertility. Imaging modalities such as TVUS may reveal indirect signs of adhesions, including distorted adnexal anatomy or hydrosalpinx. Notably, MRI can effectively distinguish between active inflammation and fibrosis, with advanced techniques improving accuracy. Fibrosis, a consequence of chronic inflammation, often shows low signal intensity on T2-weighted images and delayed or minimal enhancement after contrast administration. Techniques such as diffusion-weighted imaging (DWI) and dynamic contrast-enhanced MRI enhance this differentiation by assessing water diffusion and tissue perfusion, respectively, allowing differentiation between chronic inflammatory changes, endometriosis, and neoplasia [60,61].

Infertility is one of the most serious consequences of chronic PID. The estimated risk of infertility increases with the number of PID episodes: approximately 8% after one episode, 20% after two, and over 40% after three [62]. Tubal factor infertility is the most common form, due to loss of tubal patency, impaired ciliary motility, and peritubal adhesions. Damage to the endometrium—through chronic endometritis or altered immune response—may further compromise embryo implantation [58].

Emerging evidence suggests that chronic infections with *C. trachomatis* and *N. gonorrhoeae* may modulate host immune responses, promoting both tissue damage and immune-mediated infertility. These pathogens can evade immune clearance, persist intracellularly, and induce a chronic inflammatory microenvironment rich in pro-inflammatory cytokines and myeloid cell activation pathways, while simultaneously suppressing protective T-cell responses [57]. Histological analysis of endometrial biopsies from PID patients reveals cellular damage, fibrosis, and altered transcriptional profiles consistent with innate immune activation and impaired mucosal immunity [56].

Bacterial vaginosis and associated dysbiosis have also been implicated in the pathogenesis of chronic PID and infertility. BV-associated anaerobes such as *Gardnerella vaginalis*, *Atopobium vaginae*, and *Sneathia spp.* disrupt the normally lactobacillus-dominated vaginal microbiota, reduce mucosal barrier protection, and facilitate ascending infections [58]. The presence of BV increases the risk of both PID and tubal infertility, likely through sustained subclinical inflammation and bacterial translocation [63].

From an imaging perspective, chronic PID is associated with hydrosalpinx, tubal wall thickening, adnexal masses, and pelvic fat stranding. On MRI, DWI sequences reveal restricted diffusion in inflamed or fibrotic tissues, and apparent diffusion coefficient (ADC) values can help distinguish chronic from acute inflammation [60,61]. In a study analyzing diffusion metrics, chronic inflammation showed significantly higher ADC values compared with acute processes, indicating decreased cellularity and increased extracellular space due to fibrosis [60].

Laparoscopy remains the gold standard for diagnosing pelvic adhesions and tubal pathology. Direct visualization allows confirmation of structural damage, hydrosalpinx, and adhesions, and can guide potential surgical interventions [19,59]. However, in cases of severe tubal damage, reconstructive surgery has limited success. ART, particularly in vitro fertilization (IVF), is often the preferred option for achieving pregnancy in affected women [56].

Despite advances in imaging and treatment, prevention remains paramount. Early detection and treatment of sexually transmitted infections, coupled with screening strategies in high-risk populations, are critical to reducing the burden of chronic PID and its reproductive sequelae [62]. Preconception counseling for women with a history of PID should include fertility assessment and early referral to reproductive specialists when indicated [64] [Table 4].

## 5. Differential Diagnosis

### 5.1. Appendicitis

Appendicitis is an acute inflammation of the vermiform appendix and represents the most common abdominal surgical emergency worldwide. Its incidence is approximately 233 cases per 100,000 population annually, with a lifetime risk estimated between 6.7% and 8.6%. The age-standardized prevalence and incidence peak in the 15-to-19-year age group for both males and females [65].

Although luminal obstruction was traditionally considered the primary cause of acute appendicitis, recent evidence suggests a multifactorial etiology involving genetic predisposition, environmental influences, and infectious agents. Obstruction of the appendiceal lumen leads to increased intraluminal pressure, ischemia, and bacterial overgrowth, ultimately resulting in wall necrosis and infection.

In women of childbearing age, the differential diagnosis between PID and acute appendicitis can be challenging, as the two conditions may present with similar clinical symptoms and laboratory findings. Therefore, imaging plays a crucial diagnostic role.

Ultrasound is the first-line imaging modality for evaluating appendicitis, while CT is reserved for cases in which ultrasound is inconclusive, or it is used as the first-line imaging modality in obese or elderly patients, or when complicated appendicitis is suspected. CT findings include: increased appendix diameter (≥10 mm), thickening and stratification of the wall, wall enhancement, increased attenuation of the periappendiceal fat, thickening of the cecum, and the presence of lymphadenopathy in the ileocecal region [66].

MRI is reserved for cases with inconclusive ultrasound findings, especially in pediatric patients or pregnant women. MRI findings associated with appendicitis include an enlarged appendix (diameter ≥ 7 mm), thickening (>3 mm), and stratification of the wall, hyperintensity on T2-weighted images, and restricted diffusion of the appendiceal wall. Additional findings may include periappendiceal fat stranding, the presence of periappendiceal fluid, pericecal lymphadenopathy, and the detection of an appendicolith [67].

### 5.2. Perirectal Abscesses

Perirectal abscesses are localized collections of pus within the perirectal or perianal spaces and commonly occur as complications of Crohn’s disease (CD)**,** representing a major manifestation of perianal involvement. Perianal disease affects up to 40% of patients with CD and can be classified into two main categories: fistulizing and non-fistulizing. Fistulizing disease typically includes abscesses and fistulas, while non-fistulizing manifestations may present as hemorrhoids, skin tags, fissures, ulcers, anorectal strictures, or anorectal malignancies [68].

MRI plays a critical role in the evaluation of perirectal abscesses in Crohn’s disease and other forms of inflammatory bowel disease (IBD). Typical MRI findings include fluid collections measuring ≥10 mm in diameter, with rim enhancement following contrast administration and diffusion restriction on DWI sequences [69].

Given that PID can also result in the formation of pelvic abscesses—particularly TOAs—the imaging appearance of perianal disease in IBD, especially perirectal abscesses, should be considered in the differential diagnosis. However, unlike PID, perirectal abscesses in Crohn’s disease are often associated with perianal fistulas, and MRI typically reveals normal uterine and adnexal structures, helping to distinguish between the two conditions.

### 5.3. Endometriosis

Endometriosis is a common gynecological condition characterized by ectopic, functional endometrial glands and stroma located outside the uterus, affecting approximately 10% of women of reproductive age [70]. Clinically, it may mimic PID because of overlapping symptoms such as chronic pelvic pain, dyspareunia, and adnexal tenderness. In contrast to PID, which typically presents acutely with systemic inflammatory features, endometriosis usually follows a chronic, non-infectious course.

CT, although less sensitive than MRI for soft-tissue characterization, is frequently employed in acute settings because of its rapid accessibility. While CT lacks specificity for endometriosis, it may detect indirect findings such as retroperitoneal fibrosis or signs of deeply infiltrating disease, particularly when the bowel or urinary tract is involved. In the context of PID, CT is more likely to reveal features of acute infection, including TOAs, fat stranding, and fluid collections [26]. Nevertheless, MRI remains the imaging modality of choice for the differential diagnosis between PID and endometriosis, owing to its superior soft-tissue contrast and ability to characterize complex adnexal lesions. Both endometriomas and TOAs may present as complex adnexal masses with thickened walls and internal heterogeneity. MRI multiplanar sequences are especially helpful in identifying dilated fallopian tubes, which appear as serpentine or tubular, juxta-uterine, fluid-filled structures measuring over 5 mm in diameter. MRI also enables the detection of associated inflammatory features such as peripheral wall enhancement and complex internal content. Additional findings may include pelvic edema, thickening of the uterosacral ligaments, and stranding of the periuterine and adnexal fat. These features aid in distinguishing pyosalpinx from hydrosalpinx, a non-infectious tubal dilatation often secondary to obstruction from endometriosis or adhesions.

DWI further contributes to adnexal lesion characterization. Restricted diffusion is indicative of pyosalpinx or TOA, whereas hyperintensity on fat-suppressed T1-weighted images suggests hematosalpinx, typically caused by blood products within the fallopian tube. Hematosalpinx may occur in the context of ectopic pregnancy, adnexal torsion, endometriosis, trauma, or malignancy [11,13,24,26,71]. MRI has demonstrated high diagnostic accuracy for PID and assists in differentiating it from hydrosalpinx, endometriosis, and other mimics.

Inflammatory extension into the posterior pelvic compartment may result in uterosacral ligament thickening, a hallmark of PID readily visualized on MRI [26]. Reactive lymphadenopathy may also be observed.

In cases where diagnosis remains uncertain or symptoms are refractory to treatment, diagnostic laparoscopy continues to represent the gold standard for definitive diagnosis.

### 5.4. Oncological Pelvic Masses

Differentiating PID from oncological pelvic masses, such as epithelial ovarian carcinomas and metastatic lesions, remains a significant diagnostic challenge because of overlapping clinical features, including pelvic pain, adnexal enlargement, and elevated CA-125 levels [72]. However, CA-125 levels can be elevated in both pelvic inflammatory disease (PID) and ovarian malignancy, making it a less specific indicator for ovarian cancer, particularly in premenopausal women. TOAs, a severe complication of PID, can closely mimic ovarian malignancies on imaging. However, the presence of tubal dilation and systemic inflammatory signs supports a diagnosis of TOA, as tubal involvement is uncommon in primary ovarian malignancies [26]. The differential becomes more complex in the rare instance of primary fallopian tube carcinoma (PFTC), which accounts for less than 1% of gynecological cancers. The differential becomes more complex in the rare instance of PFTC, which accounts for less than 1% of gynecological cancers. Classical symptoms—colicky pelvic pain, a pelvic mass, and serosanguineous vaginal discharge (Latzko’s triad)—are observed in only 15% of cases, further complicating timely recognition [73].

**Figure 7:**
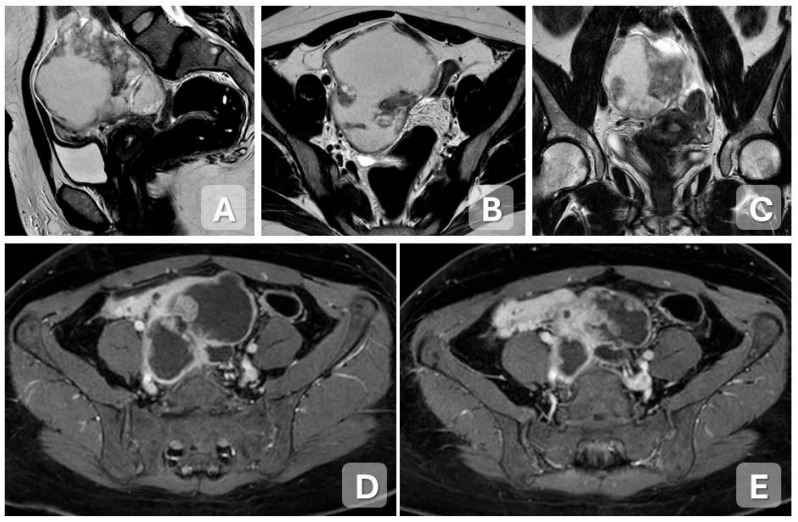

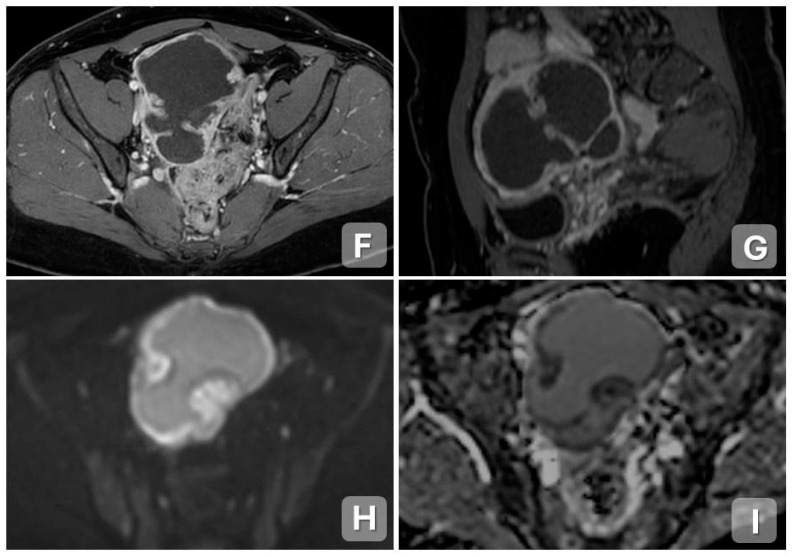
Abscessed tumor in a 35-year-old woman with fever and pelvic pain. (**A**–**C**) Sagittal, axial, and coronal T2-weighted MRI images demonstrate a complex right adnexal mass with thickened, irregular walls, internal vegetations, and heterogeneous content. (**D**–**G**) Axial and sagittal contrast-enhanced T1-weighted images show marked enhancement of the abscess wall and the solid components. (**H**,**I**) DWI and corresponding ADC map reveal pronounced diffusion restriction within the solid component, consistent with high cellularity.

CT is often the first-line imaging modality in acute or nonspecific presentations and can aid in distinguishing PID-related TOAs from malignant processes. Features favoring TOA include multiloculated adnexal masses with thick enhancing walls, pelvic fat stranding, and associated fluid collections, often accompanied by signs of systemic inflammation. In contrast, ovarian malignancies are more likely to present as solid or mixed solid–cystic masses with papillary projections, ascites, and omental caking. While CT is limited in characterizing tissue composition, its ability to assess the extent of disease and identify complications such as abscess rupture or bowel involvement is critical in the acute setting [26] [Figure 7].

MRI is a valuable tool in the evaluation of suspected PFTC. Characteristic findings include a tubular or sausage-shaped mass with solid components that exhibit T1 hypointensity, T2 hyperintensity, and moderate post-contrast enhancement [72]. Hydrosalpinx, resulting from partial tubal obstruction and secretory tumor activity, and intrauterine fluid—present in up to 30% of PFTC cases—may support the diagnosis. DWI may further support diagnosis by highlighting restricted diffusion in solid malignant components [74]. Accurate distinction between infectious and malignant adnexal pathology relies on a multimodal approach incorporating clinical, laboratory, and imaging data, with laparoscopy reserved for equivocal cases [26] [Table 5].

#### PID and Artificial Intelligence

In recent years, artificial intelligence (AI) has increasingly been integrated into medical imaging, offering valuable support in diagnosis, risk stratification, and prognostic assessment of a wide range of diseases. AI technologies, particularly those based on deep learning such as convolutional neural networks (CNNs), have demonstrated promising results across numerous clinical domains.

In gynecologic imaging, AI has been successfully applied to assess uterine fibroids, ovarian cysts, as well as in oncologic imaging [75,76], for tumor characterization, prediction of molecular profiles, and treatment response. Similarly, in abdominal imaging, models have been used to support the diagnosis and prognosis of inflammatory diseases such as acute appendicitis [77]. These represent only a few of the many applications currently being explored, as the field continues to evolve rapidly.

Despite these advances, no studies to date have specifically addressed the application of AI in imaging for pelvic inflammatory disease (PID). This remains an open and unexplored field, with significant potential given the diagnostic complexity and imaging variability of PID.

However, AI has been applied to PID through non-imaging approaches. For instance, machine learning models such as random survival forests and Cox regression have been used to predict the progression of PID to sepsis using clinical data, achieving high predictive performance [78]. A further example is the development of artificial neural network-based diagnostic support systems, which achieved high sensitivity and specificity using inputs such as clinical symptoms, laboratory data, and demographic variables—demonstrating the feasibility of AI-driven decision support for PID even in the absence of imaging data [79,80]. Additionally, in the broader context of sexually transmitted infections—common precursors of PID—AI is increasingly used to predict individual risk, monitor epidemiological trends, and guide public health strategies [81].

These examples highlight the potential of AI in PID-related diagnostics and management but underscore the current gap in studies focused on imaging applications, which could represent a valuable direction for future research.

## 6. Conclusions

PID remains a complex clinical entity with a wide spectrum of presentations, often overlapping with other gynecologic and non-gynecologic conditions. Accurate and timely diagnosis is critical, as delayed or inappropriate management can lead to severe reproductive and systemic complications. A thorough diagnostic workup is essential, including clinical assessment, laboratory investigations (with attention to inflammatory and tumor markers such as CA-125), and appropriate imaging. TVUS should be the initial imaging modality, with MRI or CT reserved for inconclusive or complicated cases. In patients with atypical features, suspected malignancy, or lack of response to empirical treatment, diagnostic laparoscopy may be warranted to establish a definitive diagnosis and guide therapy. A systematic, multimodal approach is crucial to distinguish PID from other causes of pelvic pain and adnexal masses, enabling timely, targeted management and improved clinical outcomes. Ultimately, for complex cases of pelvic inflammatory disease (PID), a follow-up imaging strategy should include early and regular monitoring, particularly with ultrasound or CT scans. Follow-up imaging is recommended every few days to weeks, depending on the severity and response to treatment, to assess complications and to track treatment response to therapies.

## Figures and Tables

**Table 1:** Imaging study results in pelvic inflammatory disease (PID).

	Modality	Authors	Title	Patients	Aim of Study	Sensitivity (%)	Specificity (%)
1	TVUS + Doppler	Molander P. et al., 2001 [5]	Transvaginal power Doppler findings in laparoscopically proven acute pelvic inflammatory disease	Not stated	To assess the utility of power Doppler ultrasound in detecting inflammatory hyperemia in laparoscopically confirmed PID	Not specified	Not specified
2	TVUS	Romosan G., Valentin L., 2014 [6]	The sensitivity and specificity of transvaginal ultrasound with regard to acute pelvic inflammatory disease: a review of the literature	Review	To summarize the diagnostic performance of TVUS in PID	~80 (range 71–90)	~85 (range 77–95)
3	CT	Lee M.H. et al., 2014 [7]	CT findings of acute pelvic inflammatory disease	Not stated	To describe key CT findings in acute PID	Descriptive	Descriptive
4	CT	Jung S.I. et al., 2011 [10]	Acute pelvic inflammatory disease: Diagnostic performance of CT	55	To evaluate the diagnostic performance of CT in patients with PID	78.6	83.3
5	CT	Revzin M.V. et al., 2016 [11]	Pelvic Inflammatory Disease: Multimodality Imaging Approach with Clinical-Pathologic Correlation	Not stated	To illustrate imaging features of PID with different modalities	Descriptive	Descriptive
6	MRI	Tukeva T.A. et al., 1999 [12]	MR imaging in pelvic inflammatory disease: comparison with laparoscopy and US	34	To compare MRI with laparoscopy and ultrasound in suspected PID	95	89
7	MRI	Li W. et al., 2013 [14]	Pelvic inflammatory disease: evaluation of diagnostic accuracy with conventional MR with DWI	61	To determine the added value of DWI in MRI for diagnosing PID	98.4 (with DWI)/90.7 (without DWI)	97.5 (with DWI)/91.2 (without DWI)

**Table 2:** Imaging key points.

**Transvaginal Ultrasound (TVUS)**
First-line imaging modality for suspected PID due to availability, low cost, and good patient tolerability.
High-frequency transducers (5–9 MHz) provide excellent resolution of the uterus, adnexa, and pelvic spaces. Doppler imaging enhances diagnostic confidence by revealing inflammatory hyperemia.
3D ultrasound may aid in differentiating tubo-ovarian abscesses (TOA) from neoplastic masses.
Limitations include operator dependency, discomfort in acute pain, limited field of view, and reduced sensitivity in early or atypical presentations.
Diagnostic accuracy may be overestimated in the literature, as most comparative studies involve advanced-stage disease confirmed via laparoscopy.
**Computed Tomography (CT)**
Second-line tool, particularly useful in emergency settings or when TVUS is inconclusive.
Findings include fallopian tube thickening, pelvic fat stranding, endometrial/uterine enhancement, and complex adnexal masses.
Not recommended as first-line imaging due to ionizing radiation and suboptimal soft-tissue contrast.
Evidence base largely comprises retrospective and small-scale studies.
**Magnetic Resonance Imaging (MRI)**
High diagnostic performance with reported sensitivity and specificity up to 98.4% and 97.5%, respectively, especially when using diffusion-weighted imaging (DWI).
Comprehensive protocol includes T1- and T2-weighted sequences, fat suppression, post-contrast imaging, and DWI.
Particularly valuable in complex, indeterminate, or inconclusive cases to avoid unnecessary invasive procedures.
Limitations include high cost and limited availability hinder routine first-line use.

**Table 3:** Acute pelvic inflammatory disease keypoints.

**Salpingitis and Pyosalpinx**
**Ultrasound** Early sign: **tortuous, thickened tube (>5 mm)** with fluid. **Hydrosalpinx**: anechoic tube without wall thickening; often chronic. **Pyosalpinx**: echogenic fluid and debris within dilated tube; **“Cogwheel”** and **“Beads-on-a-string”** signs are diagnostic. **Color Doppler**: increased vascularity in active inflammation.
**MRI:** Pyosalpinx: mixed T1/T2 signal; wall enhancement and surrounding fat stranding. **DWI**: restricted diffusion in purulent material improves diagnostic accuracy. Post-contrast enhancement reflects acute inflammation; involvement of the parametrial and pelvic fascial planes may indicate disease spread.
**CT**: Nonspecific but helpful in emergency settings to rule out mimics (appendicitis, diverticulitis). Useful for detecting complications (abscess, bowel involvement).
**TOA**: complex adnexal mass with solid–cystic components, septations, hyperechoic foci (gas/debris).
**MRI**: Abscesses: **T1 hyperintense** if hemorrhagic/proteinaceous; **T2 hypointense.** Marked wall enhancement, pelvic fat stranding, thickened uterosacral ligaments, and free fluid suggest severe disease.
**CT:** Useful in emergency or suspected **TOA rupture**, peritonitis, or bowel involvement. Abscesses show hypodense (fluid) and hyperdense (debris, hemorrhage) areas ± gas bubbles.
**Tubo-Ovarian Complex (TOC) and Tubo-Ovarian Abscess (TOA)**
**TOC**: inflamed, adherent ovary and fallopian tube remain separately identifiable. **TOA**: indistinct adnexal structures due to confluent abscess formation; may require hospitalization.
**Ultrasound**: complex mass with cystic and solid areas, septa, debris, gas, hyperemia.
**MRI**: peripheral wall enhancement, T1 hyperintensity (if hemorrhagic), T2 hypointensity centrally; ancillary signs include fascial enhancement and ligament thickening
**Pelvic Peritonitis**
Advanced PID stage with peritoneal involvement. Loculated/complex fluid collections. Peritoneal enhancement and pelvic fat stranding. Bowel wall thickening, displacement, and pneumoperitoneum in advanced cases.

**Table 4:** Chronic pelvic inflammatory disease keypoints.

**Pathogenesis**
Results from unresolved, recurrent, or subclinical infections (notably *C. trachomatis*, *N. gonorrhoeae*, and *M. genitalium*).
Leads to tubal scarring, peritoneal adhesions, and altered immune response.
**Clinical Sequelae**
Chronic pelvic pain, dyspareunia, and infertility.
**Imaging**
Transvaginal Ultrasound (TVUS): May reveal indirect signs of chronic PID, such as distorted adnexal anatomy and hydrosalpinx. Limited in detecting pelvic adhesions directly.
Magnetic Resonance Imaging (MRI): Superior soft-tissue contrast and multiplanar capability. Tubal wall thickening, adnexal masses, and pelvic fat stranding.
**Diffusion-weighted imaging (DWI)** improves lesion characterization.
Helps differentiate **chronic inflammation, endometriosis**, and **neoplasia**. **Apparent diffusion coefficient (ADC)** values tend to be **higher** in chronic inflammation due to fibrosis (lower cellularity, increased extracellular space).
**Imaging vs. Direct Visualization**
Imaging is crucial for non-invasive assessment, pre-surgical planning, and monitoring disease progression. Laparoscopy remains the gold standard for diagnosing: pelvic adhesions, tubal occlusion, and hydrosalpinx.
**Clinical Implications and Infertility**
The risk of tubal factor infertility increases with repeated PID episodes: 8% after one, 20% after two, and 40% after three episodes.

**Table 5:** Differential diagnosis of PID: Imaging-focused insights.

**Appendicitis**
Ultrasound (US) is the first-line modality, with CT reserved for inconclusive US, obese/elderly patients, or suspicion of complications. CT signs suggestive of appendicitis: appendix diameter ≥ 10 mm, mural thickening and stratification, cecal wall thickening, periappendiceal fat stranding, and regional lymphadenopathy. MRI, preferred in pregnancy and pediatrics, reveals: appendix diameter ≥ 7 mm, wall thickening > 3 mm, T2 hyperintensity, restricted diffusion, periappendiceal inflammation, and possible appendicolith.
**Perirectal Abscesses (Crohn’s Disease)**
Perirectal abscesses are frequent in fistulizing Crohn’s disease, affecting up to 40% of CD patients. MRI is the gold standard for detecting perirectal abscesses in IBD: key findings include fluid collections ≥ 10 mm, rim enhancement, and diffusion restriction on DWI. Differentiation from PID is aided by the presence of perianal fistulas and normal adnexal/uterine structures on MRI, absent in PID.
**Endometriosis**
Chronic pelvic pain and adnexal tenderness can mimic PID, but endometriosis lacks systemic inflammatory signs and follows a chronic course. CT is frequently used in emergency settings but is nonspecific for endometriosis; it may reveal signs of deeply infiltrating disease. MRI is highly accurate in distinguishing PID from hydrosalpinx and endometriosis, reducing unnecessary interventions. MRI is the modality of choice because it detects endometriomas, dilated serpentine fallopian tubes (>5 mm), complex adnexal masses, peripheral wall enhancement, uterine ligament thickening, and fat stranding.
DWI enhances differentiation: Restricted diffusion = pyosalpinx or TOA (PID). T1 hyperintensity = hematosalpinx (often endometriosis-related).
**Oncological Pelvic Masses**
TOAs (complications of PID) can mimic ovarian malignancy, especially epithelial ovarian carcinoma or primary fallopian tube carcinoma (PFTC). Differentiation requires a multimodal approach: clinical features, labs (e.g., CA-125), and imaging.
CT is typically the first imaging tool; TOAs are multiloculated with thick enhancing walls, fat stranding, and inflammatory fluid collections.
MRI supports characterization: PFTC findings: sausage/tubular masses, T1 hypointense, T2 hyperintense, moderate enhancement, hydrosalpinx, intrauterine fluid. DWI reveals restricted diffusion in solid malignant components.

## Abbreviations

ADC: apparent diffusion coefficient; ART: assisted reproductive technologies; CD: Crohn’s disease; CT: computed tomography; DWI: diffusion-weighted imaging; FHCS: Fitz-Hugh–Curtis syndrome; IBD: inflammatory bowel disease; IUD: intrauterine device; IVF: in vitro fertilization; MDCT: multidetector computed tomography; MRI: magnetic resonance imaging; PFTC: primary fallopian tube carcinoma; PID: pelvic inflammatory disease; RPOC: retained products of conception; STIs: sexually transmitted infections; TOA: tubo-ovarian abscess; TOC: tubo-ovarian complex; TVUS: transvaginal ultrasound.
